# Using Deep Learning with Thermal Imaging for Human Detection in Heavy Smoke Scenarios

**DOI:** 10.3390/s22145351

**Published:** 2022-07-18

**Authors:** Pei-Fen Tsai, Chia-Hung Liao, Shyan-Ming Yuan

**Affiliations:** Department of Computer Science, National Yang Ming Chiao Tung University, Hsinchu 300, Taiwan; alicetsai.cs10@nycu.edu.tw (P.-F.T.); aiallen.cs07g@nctu.edu.tw (C.-H.L.)

**Keywords:** thermal imaging camera, LWIR, infrared thermal camera, convolutional neural network, evacuation in fire, human detection, smoky fire scene, firefighter protection, human rescue, YOLO, real-time object detection

## Abstract

In this study, we propose using a thermal imaging camera (TIC) with a deep learning model as an intelligent human detection approach during emergency evacuations in a low-visibility smoky fire scenarios. We use low-wavelength infrared (LWIR) images taken by a TIC qualified with the National Fire Protection Association (NFPA) 1801 standards as input to the YOLOv4 model for real-time object detection. The model trained with a single Nvidia GeForce 2070 can achieve >95% precision for the location of people in a low-visibility smoky scenario with 30.1 frames per second (FPS). This real-time result can be reported to control centers as useful information to help provide timely rescue and provide protection to firefighters before entering dangerous smoky fire situations.

## 1. Introduction

Fire is one of the biggest workplace safety threats. It is a hazard that we would want to not only prevent in advance, but also to respond to quickly in terms of emergency response. An effective fire evacuation plan is important to save human lives, protect firefighters, and minimize property loss [1]. We will focus on the application of the YOLOv4 model with a thermal camera following the National Fire Protection Association (NFPA) 1801 [2] standards to detect humans in a smoky fire scene during an emergency evacuation.

It is widely known that the most dangerous factor in a fire scene is smoke and heat, especially smoke. Smoke causes zero visibility and people can die from smoke inhalation during evacuations from buildings or from firefighter rescue [3].

In a fire situation, smoke spreads at a speed of 3–5 m/s, while humans’ top movement speed is 0.5 m/s. Where the smoke arrives, the fire will follow. With this in mind, how to locate people and guide them out of a heavy smoke environment will be the key to surviving an evacuation process. Detecting a human in heavy smoke is a challenging task. The most commonly used solutions are laser detection and ranging (LADAR), 3D laser scanning, ultrasonic sensor, or infrared thermal camera [3,4].

Here, we propose to use an infrared thermal camera that follows NFPA1801 standards for good visibility in a smoky fire environment with the YOLOv4 [5] artificial intelligence human detection model as a convolutional neural network (CNN) processed by a single GPU computer to locate humans in a smokey scene, while providing information to the central control room during an emergency evacuation.

## 2. Background and Related Work

### 2.1. Thermal Spectrum and Sensor

**Figure 1:**
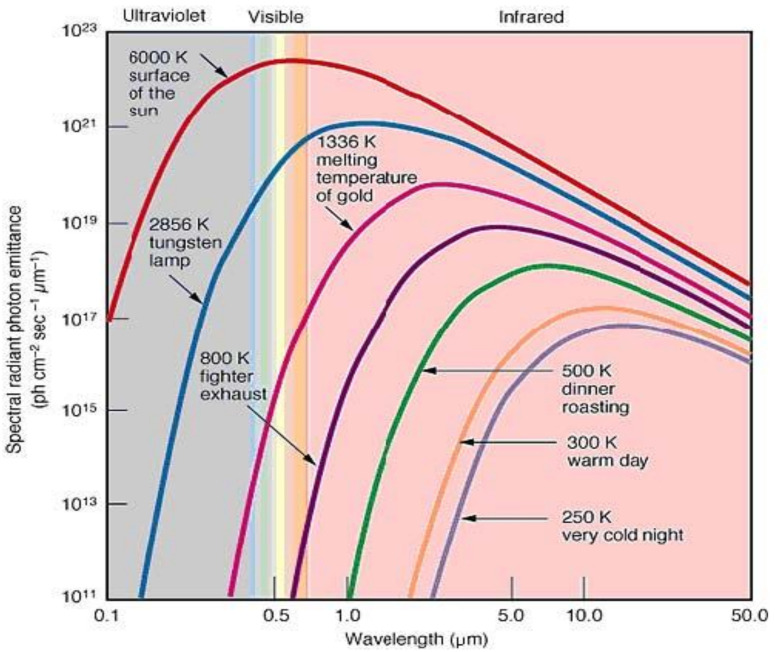
Thermal radiation in different temperatures (Hajebi, 2008; pp. 105–112) [6].

Objects above absolute zero emit infrared radiation across a spectrum of wavelengths referred to as thermal radiation, as shown in Figure 1. The higher the thermal radiation, the shorter the wavelength in the spectrum.

A thermal imaging camera (TIC) converts the thermal radiation into an electric signal to make the invisible infrared (IR) wavelength into a visible RGB or grayscale image. This is a commonly used approach to detect heat sources and to change temperatures into a visible digital image.

**Figure 2:**
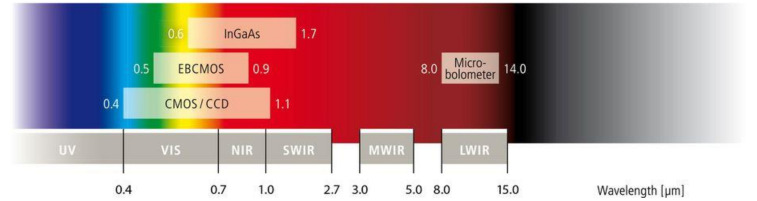
Camera sensor type responding to different wavelengths.

IR from 0.7 μm to 15 μm is invisible to the human eye. Different sensor materials can be used to detect corresponding wavelength ranges, as illustrated in Figure 2 [6]. A micro-bolometer is commonly used as a long-wavelength infrared (LWIR) TIC since it does not require low temperature cooling and provides good contrast with human images in both day and night as described in Table 1.

### 2.2. Different Sensor Used in Smoky Fire Scene

A variety of instruments have been studied that can be used in fire and smoky situations, including visible camera, kinetic depth sensor, LIDAR, night vision, IR camera, radar, and sonar. It has been demonstrated that the most efficient instruments are thermal cameras, radar, and LIDAR.

The time-of-flight technology of LIDAR and radar have the best distance measurement accuracy that is suitable for robot navigation [6,7,8,9].

Thermal cameras and radar are the best technology to penetrate heavy smoke and are less affected by heavy smoke and high temperatures.

### 2.3. NFPA1801 Standard of Thermal Imagers

The National Fire Protection Association (NFPA) defines a consensus standard for the design and performance of thermal imaging sensors (TICs) to be used in smoky fire scenarios [10]. The NFPA defines the standard for TICs because the high-temperature and low-visibility conditions are dangerous for firefighters. It defines the criteria for TICs including “interoperability”, “durability”, and “resolution” to make it easy to operate the Tic in a smoky fire scene.

The interoperability is to allow firefighters to operate a TIC with no hesitation during a rescue to save both victims and themselves in a low-visibility scene.

The durability is to enable the TIC to operate in a high-flame or dusty environment with no malfunction.

**Figure 3:**
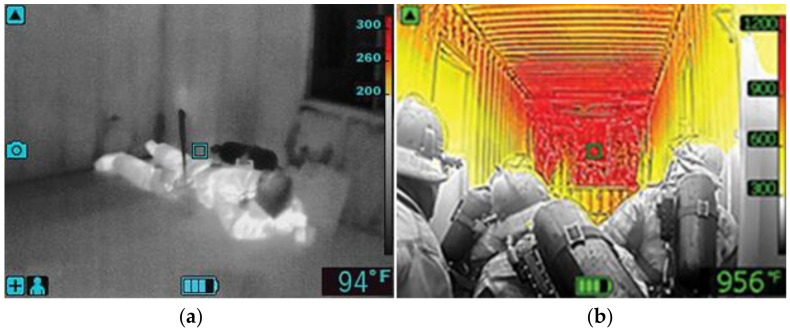
Different sensitivity image comparison (**a**) Medium sensitivity: 0 °F–300+ °F (**b**) Low Sensitivity: 0 °F–1200 °F (Source: TechValidate. TVID: B52-065-90D) [11].

The most important specifications for TICs on the market for various applications include:

- High resolution (>320 × 240),
- High refresh rate (>25 Hz),
- Wide field of view,
- Low temperature sensitivity (0 °F–650 °F),
- Temperature bar: gray, yellow, orange, and red (Figure 3).

Section 6.6.3.1.1 indicates the low sensitivity mode indicator as a basic operational format. The intention of this design is to show high-heat regions in color to enable firefighters to easily distinguish dangerous areas, and to present victims in gray for easy searching, as in Figure 3.

**Figure 4:**
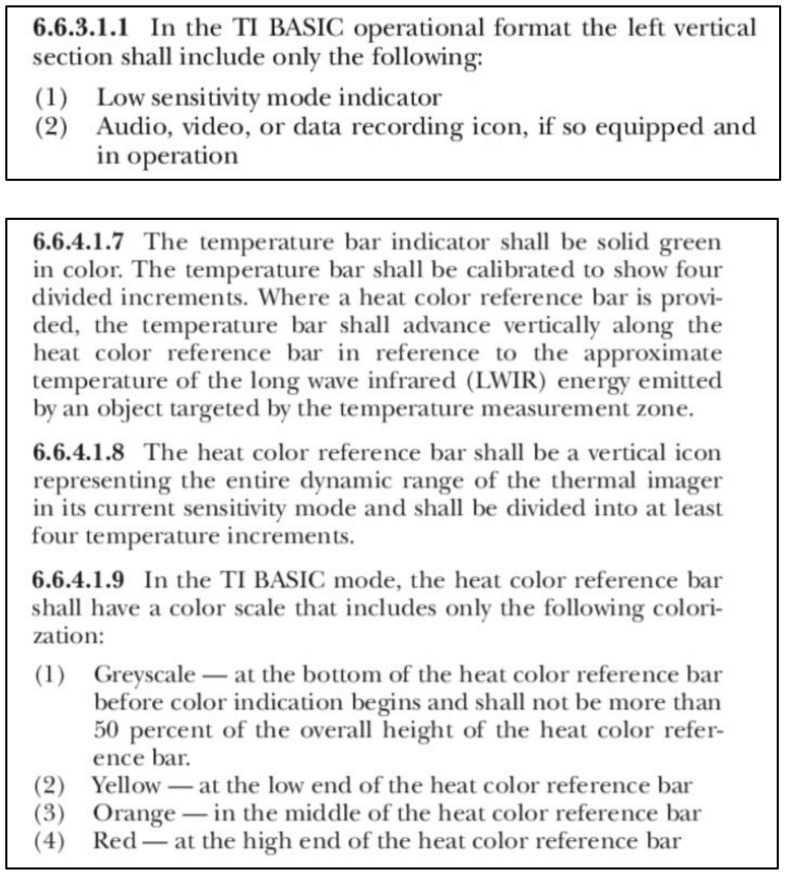
Definition of NFPA1801 for TIC sensitivity and temperature colorization bar [10].

**Figure 5:**
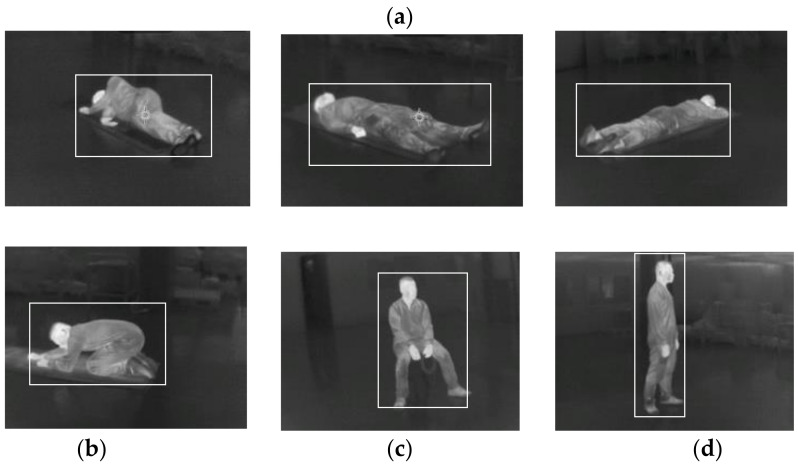
Flue Ti300+ self-taken images to include (**a**) side lying, lying upward, lying downward, (**b**) squatting, (**c**) sitting, and (**d**) standing postures.

Section 6.6.4 defines the temperature in the color bar to have <50% temperature range in gray and higher temperatures in yellow, orange, and red, respectively, as in Figure 4. TICs that follow NFPA1801(2021) on the market for firefighting purposes commonly define the 25% temperature range in gray, as in Figure 5. In this case, the 25% range for gray will be 162.5 °F (72.5 °C), meaning that humans fall within the grayscale at the 105 gray level, as in Figure 4.

NFPA code 1801: Standard on Thermal Imagers for the Fire Service, 2021 edition. In NFPA National Fire Codes Online. Retrieved from http://codesonline.nfpa.org.

### 2.4. Different Sensors Used in Smoky Fire Scenes

There is an increasing number of papers demonstrating the ability of firefighting robots with stereo infrared vision, radar, or LIDAR to generate fusion images or environmental measurements to identify and locate objects [8,9,12].

In low-visibility smoky scenes, robots can be implemented for environment mapping and indoor navigation. Among the studies on this topic, 3D infrared vision is always implemented into the system that can be used for distance measurement and object detection.

There is related work using FLIR imaging that followed NFPA1801 with a CNN model to help firefighters to navigate in a fire scene [13]. It shows the capability of convolutional neural network models for detecting humans with a thermal imaging camera.

### 2.5. Convolutional Neural Network (CNN) Object Detection

The task of object detection requires the localization of the objects of interest with coordinates or a bounding box in the image frame.

The CNN model has robust object detection results compared to the traditional computer vision model [14], because of the benefit of the feature extraction of a full image by sliding windows and the combination of multi-scale feature extraction with the increasing depth of network architecture for complex features.

Successful CNN architectures have been proposed recently for object detection tasks, such as R-CNN [15], RFCN [16], Fast R-CNN [17], Faster R-CNN, Mask R-CNN [18], and YOLO [19].

YOLOv4 has been shown to be a fast, real-time object detection technique with high accuracy, with a precision of 43.5% in MS COCO datasets and fast detection of 65 FPS (frame per second) with Nvidia GPU Tesla V100 [5].

There is also evidence of an LWIR sensor with a faster R-CNN model in military surveillance [20] achieving a mean average precision (mAP) of 87% in military operations. In outdoor pedestrian detection, a region-CNN-based model is proposed to achieve an mAP of 59.91% [21].

## 3. Experiment Methodology

### 3.1. Model Fluke Ti300+ of Thermal Imaging Camera (TIC) for Data Collection

The model specifications for the Fluke Ti300+ are listed in Table 2. This model was chosen for thermal image data collection since it follows NFPA1801 in terms of resolution, temperature sensitivity, and spectral range with an uncooled microbolometer.

We use this TIC to generate extra postures of people falling, lying, and squatting in 360° as training data for the scenario of people requiring help in the event of an evacuation in a smoky environment, as in Figure 5. Human body temperature will correspond to a GL of 105, which is the same gray level as our self-taken human thermal image.

### 3.2. Thermal Datasets for Training

**Figure 6:**
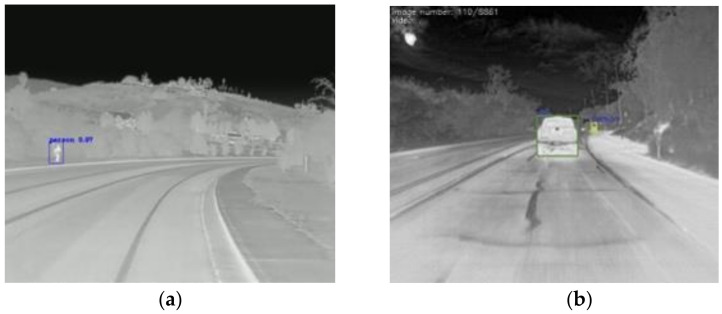
FLIR ADAS thermal image for walking people and cars on the road (**a**) people walking on the road marked with blue box, (**b**) Car driving on the road with green box. Teledyne FLIR LLC (2022) [22].

**Figure 7:**
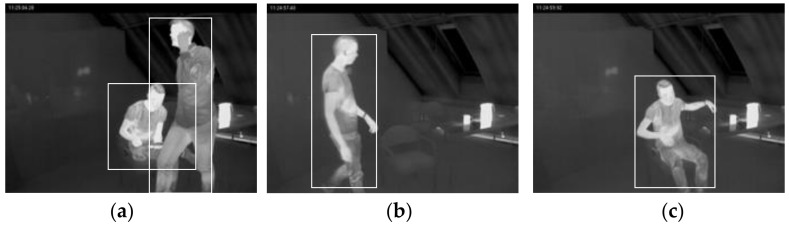
Kaggle AAU thermal indoor image of (**a**) people sitting and standing in one scene, (**b**) walking and (**c**) sitting posture [23].

The ‘V’s of big data: volume, variety, velocity, and veracity are important factors for deep learning. There is a problem of lack of volume in public thermal image datasets. In this case, we combine our self-taken images, as in Figure 5, and other thermal datasets including pedestrians as in Figure 6, and the Kaggle dataset of indoor people, as in Figure 7, to increase the volume and variety of the training data in Table 3.

### 3.3. YOLOv4 Model

In [24], it is proven that the YOLO Darknet 2.0 is capable of detecting normal and tiny long-range thermal objects, since thermal images are normally limited to low resolution and tiny objects are only 50 pixels in area. The deep network of Darknet is capable of adding values to thermal image applications in more scenarios.

We use the YOLOv4 [5] model as a CNN-based object detector, which is the highest real-time object detection model in 2020 that fits our scenario of human detection in a hazardous environment.

The model is trained on a single conventional 2080 GeForce GPU as a real-time detector of below the backbone, neck, and head. With deep networks, the backbone is for a different scale of feature extraction, the neck with SPP is for reducing model parameters for training efficiency, and the head of YOLOv3 is for one-stage classification and localization.

- **Input:** Image
- **Backbone:** CSPDarknet53 [25]
- **Neck:**
  - Additional blocks: SPP [26]
  - Path-aggregation blocks: PAN [27]
- **Head:**
  - Dense Prediction(one-stage): YOLOv3 [28]
- **Selection of BoF and BoS:**
  - Activation: Mish [29]
  - Bounding box regression loss: CIoU
  - Data augmentation: DropOut
  - Normalization: Batch Normalization [30]

The input image resolution is set to 608 × 608 for better detection of small, occluded, or overlapping humans into accurate objects classification and localization.

The pre-trained weight of COCO datasets from the GitHub of AlexeyAB/darknet [31] is used as a good approach to give a good initial position and fast converge within 5000 epochs.

The data augmentation of saturation factor 1.5 is used to cover the different brightness of scenes due to limited available training data volume.

### 3.4. Optimal Model Selection

We include three datasets of FLIR ADAS, Kaggle AAU TIR image, and Fluke Ti300+ self-taken 360-degree thermal images, as in Figure 7, and with a self-labeled bounding box as the people class. All images are in LWIR (8–15 μm) as it has the best image visibility in the IR spectral range.

The model was trained to 5000 epochs with a learning rate of 0.0014 and a burn-in of 1000, which is less than one day in our system with default hyperparameters, since the thermal human image is of good contrast and extracting features at various scales with 53 layers of network is quite efficient.

**Figure 8:**
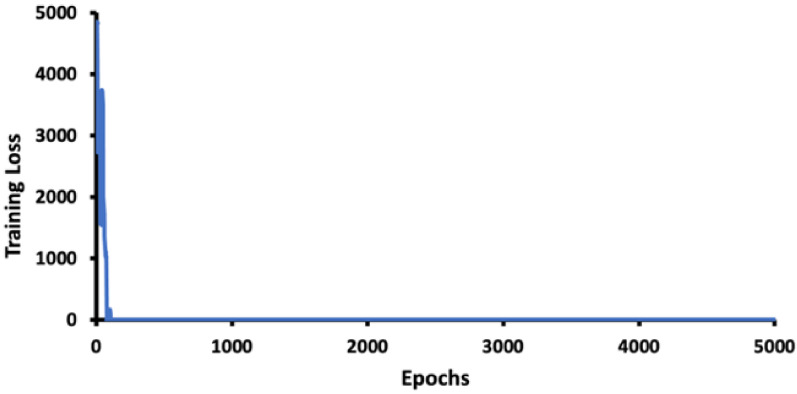
Training loss in epochs.

Figure 8 shows that there was a very fast loss drop after 1000 epochs and convergence within 5000 epochs.

**Figure 9:**
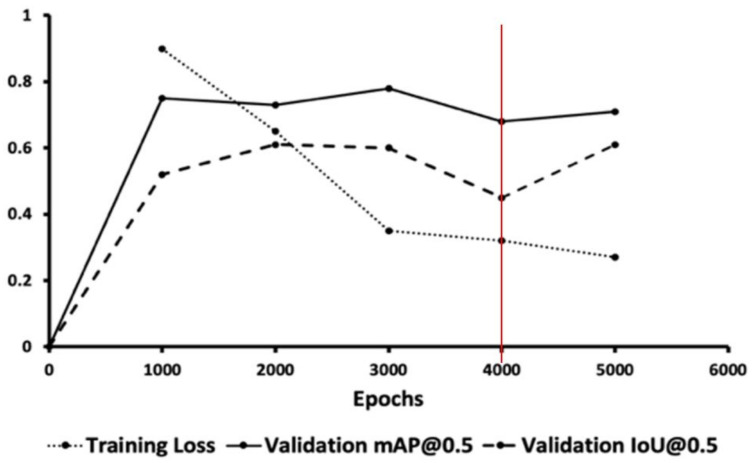
Graph of training loss, validation mAP (mean average precision) and IoU (intersection of union) and red line is the optimal weight with lowest training and validation loss, and highest mAP.

To prevent overfitting due to the small training data volume, we used validation set accuracy for optimal weights selection at 4000 epochs for our model, as shown in Figure 9. The optimal model can be trained within 24 h on a single GPU to achieve model convergence and to avoid overfitting of the training set.

## 4. Experiment Result

### 4.1. Ground Truth Calculation of Occluded Objects

In our scenario, if a person is occluded, we still want to detect and count them as one independent object. However, if two people are too close to each other, and one is occluded by the other for over 50%, then this counts as one object.

**Figure 10:**
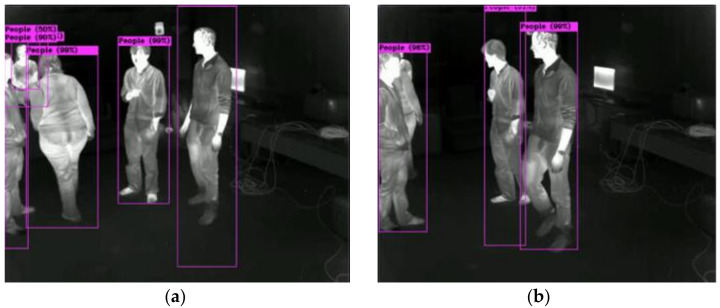
Ground truth for occluded objects: (**a**) five objects and (**b**) three objects.

For instance, as illustrated in Figure 10, the left image will count five people as ground truth (GT), while the right image will count three people as GT, since on the right side, three people are too close together to be treated as individuals.

This ground truth is used for calculating the intersection of union (IoU) of localization precision as true positive (TP) or false negative (FN). We combine the IoU and classification for the object detection index of precision, recall, and PR curve for our model efficiency.

### 4.2. Metric for Precision, Recall, and Accuracy

We followed the metric of PASCAL VOC for object detection accuracy definition as IoU ≥ 50% and correct classification as true positive, IoU < 50% missing detection as false negative, and bounding other objects as human as false positive, as in Table 4.

### 4.3. Precision, Recall, and PR Curve in Test Datasets

We chose weights at 4000 epochs as the best weights for our model with the lowest training loss, but high accuracy and object localization with the test dataset described in Table 5.

**Figure 11:**
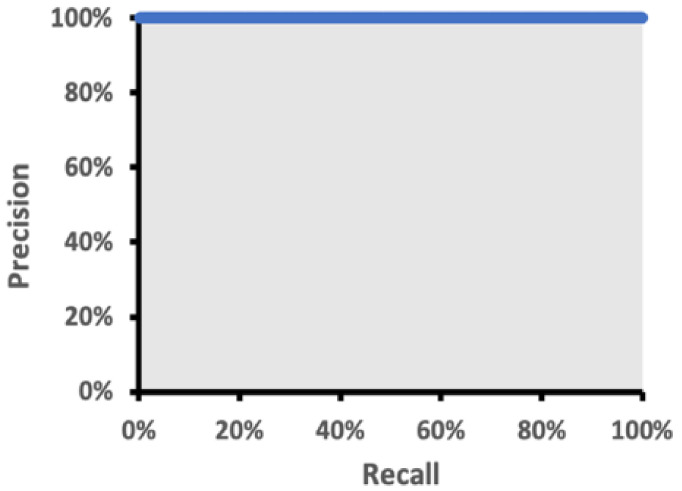
Precision to recall curve.

We achieve a detection of >97% in both precision and recall in Table 6, and the curve in Figure 11 shows an ROC > 98% with various kinds of human postures and viewing angles. This is the power of 53 layers of deep CNN network modeling, resulting in a precise bounding box of >50% IoU.

### 4.4. Detection of People in Different Postures

#### 4.4.1. Standing

**Figure 12:**
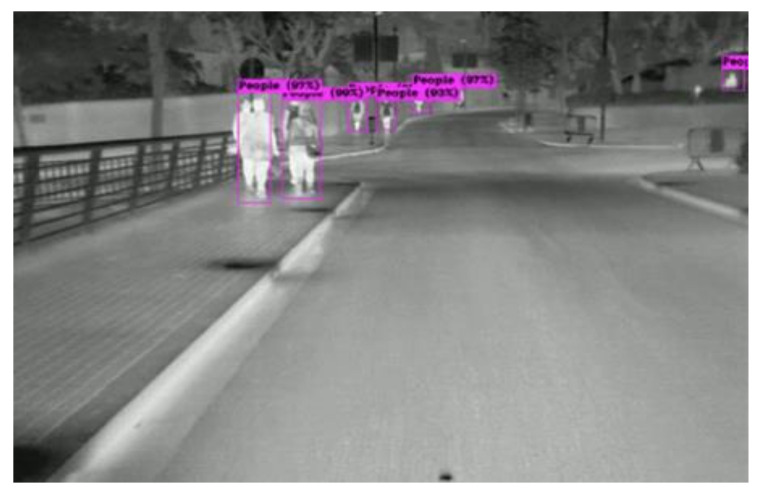
Detection of walking people in CVC dataset.

For the standing posture, our model achieves very high precision, since the FLIR training data have small and large sizes of pedestrians and the PAN implementation in the neck structure for feature aggregation. In Figure 12, the infrared images from CVC [18] demonstrate the ability for large and small human detection. Moreover, the detection of a person who is 50% occluded gives an IoU > 50%, and will be useful to replace human eye searching in the scene.

#### 4.4.2. Sitting

**Figure 13:**
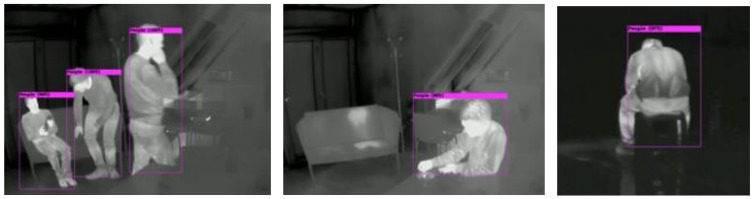
Detection of ready-to-sit, sitting, occluded sitting human, and human sitting backwards.

From the testing data from Kaggle AAU Scene 3 in Figure 13 as an indoor sitting test image, our model can detect the ready-to-sit and sitting posture with over 90% confidence; even an occluded sitting human can be detected with 99% confidence. In addition, an image of a sitting person with no face can be detected and labeled correctly with no problems.

#### 4.4.3. Lying

**Figure 14:**
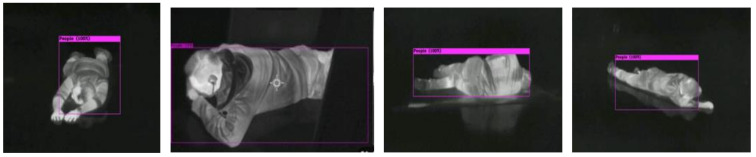
Detection of human lying on the floor from different viewing angles.

A lying posture is the most challenging task, despite being the most important posture that we want to detect in an emergency evacuation scenario. In Figure 14, our model shows >80% confidence of detection, even with the human body occluded.

In a scenario in which the person has fainted and is lying on the floor, it means they are not able to leave the hazard by themselves and require help from others. People lying down on the floor should be the top priority for rescue.

#### 4.4.4. Squatting

**Figure 15:**
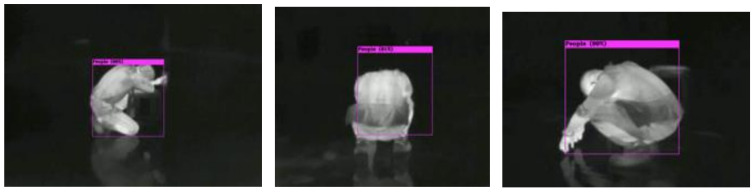
Detection of squatting human from different viewing angles.

The squatting posture of a human, as shown in Figure 15, including left side, backside, and right side, can be detected successfully, while the confidence level will drop 5% and the bounding box shift 10%. This gives a hint that training datasets are not large enough and need to try some data augmentations or more squatting images to improve the dataset variants.

### 4.5. FPS (Frame per Second) for Real-Time Test

The accurate localization and real-time detection of humans are two key factors for rescuing people in danger. This is the reason that we chose one stage object detection of YOLOv4 as our model proposal. In [5], YOLOv4 achieves a state-of-the-art result of 65FPS and 43.5% AP (65.7%AP50) in MS COCO datasets with Tesla V100, published in 2020.

This paper demonstrates the capability for the real-time object detection of a deep learning model on an embedded system of NVIDIA Jetson as a firefighter’s protective equipment (PPE) [32]. The benefit of an embedded system is small volume and low power consumption.

We compared the FDS with same YOLOv4 model on an NVIDA GeForce series GPU and Jetson embedded system. On the NVIDIA GeForce RTX2070 with 8GB memory, YOLOv4 achieves 40FPS with an input resolution of 608 × 608, which is a similar FPS to this paper of 55FPS with 416 × 416 input resolution. The Jetson Nano has only 1.1 FPS, as shown in Table 7, which is a similar FPS to the Jetson TX2 in paper [33]. We found that there is still a gap in terms of conducting real-time detection on a Jetson Nano-embedded system.

## 5. Discussion

One of the ways to achieve the real-time detection of YOLOv4 on a GeForce GPU will rely on a firefighting robot. There are some studies related to firefighting robots [7,9,12] with thermal imaging cameras to enable robots to build the map and search for people in the building during an evacuation, and send back the information regarding the locations of people to provide timely help.

The other way is to reduce the model size to fit the relatively low computation capability of the embedded system. In this way, the trade-off of a shallower model is less accuracy for human localization.

## 6. Conclusions

We successfully demonstrate the YOLOv4 deep learning model’s capability and benefit for detecting humans in heavy smoke using a thermal imaging camera that follows NFPA1801, with high resolution and low temperature sensitivity, that sufficiently enhances humans as grayscale in high-temperature and low-visibility fire scenes.

Using MS COCO pretrained weights and default parameters, the model can converge within 4000 epochs in one day’s training on single Nvidia GeForce 2070 GPU. The accuracy with an IoU of 50% can achieve >95% for the standing, sitting, lying, and squatting postures of humans; even those occluded by 50% can be detected as individual objects. This would be useful for evacuation scenes with people in crowds for counting and searching for people who require help. Real-time detection with 30.1 frames per second is achieved by a trained YOLOv4 model.

In the future, this approach can be combined with robot fusion detection [7,9,12] to allow robots to build the environment map and carry out the search for humans in buildings under evacuation, and send back the information of human locations to provide timely help. The heat source location can also be sent back to the information center to provide useful information before firefighters enter dangerous situations.

## Figures and Tables

**Table 1:** Spectrum wavelength range and properties.

Spectrum	Wavelength Range	Property
Visible spectrum	0.4–0.7 μm	Range visible to the human eye.
Near-infrared (NIR)	0.7–1.0 μm	Corresponds to a band of high-atmosphere transmission, yielding the best imaging clarity and resolution.
Short-wave infrared (SWIR)	1–3 μm	
Mid-wave infrared (MWIR)	3–5 μm	Nearly 100% transmission, with the advantage of lower background noise.
Long-wave infrared (LWIR)	8–15 μm	Nearly 100% transmission on the 9–12 μm band. Offers excellent visibility of most terrestrial objects.
Very long-wave infrared (VLWIR)	>15 μm	

**Table 2:** Specification of Fluke Ti300+ with IR-fusion technology.

Functionality	Specification
Resolution	320 × 240 pixels
Temperature Range	−20 °C–650 °C (−4 °F–1212 °F)
Spectral Range	7.5 µm–14 µm

**Table 3:** Training dataset.

Datasets	Resolution	Objects in Scene	Main Posture	# of Frames			Environment
				With Person	w/o Person	Total	
FLIR ADAS	640 × 480	People, Car, Bicycle	Walking, Standing	70	830	900	Street
KAGGLE AAU	640 × 512	People, Table,	Sitting	450	0	400	Indoor
FLUKE TI300 SELF-TAKEN	320 × 240	People	Lying, Squatting	450	0	450	Dark Room

**Table 4:** Precision and recall definition.

	Real Label			
**Predicted**		People Labeled	People Not Labeled	
	People Labeled	TP GT with IoU ^1^ ≥ 0.5	FP BG is labeled as People	Precision = TP/# Predicted
	People Not Labeled	FN GT with IoU ^1^ < 0.5	TN BG is not labeled as People	
		Recall = TP/# GT		

^1^ IoU: intersection of union.

**Table 5:** Test dataset.

Datasets	Resolution	Objects in Scene	Main Posture	# of Frames			Environment
				With Person	w/o Person	Total	
PTB-TIR CLASSROOM	640 × 480	People, Monitor	Walking, Standing	450	103	553	Classroom
KAGGLE AAU	640 × 512	People, Table,	Sitting	100	40	140	Indoor
FLUKE TI300 SELF-TAKEN	320 × 240	People	Lying, Squatting	369	0	369	Dark Room

**Table 6:** Precision and recall in test dataset.

Property	Standing	Sitting	Lying	Overall
Recall	98.05%	100%	98.64%	98.52%
Precision	100%	100%	100%	100%

**Table 7:** Comparison FPS ^1^ of Nvidia GeForce and Jetson series.

	Nvidia GeForce RTX 2080Ti	Nvidia GeForce RTX 2070	Nvidia Jetson Nano	Nvidia Jetson TX2
Computation Capability	72 TFLPS ^1^	52 TFLPS	0.5 TFLPS	6 TFLPS
Inference Efficiency	40 FPS ^2^ [33]	30.4 FPS	1.1 FPS	2.1 FPS [33]
Power Consumption	285 W	175 W	5–10 W	10–15 W

^1^ FLPS: floating-point operation per second; ^2^ FPS: frames per second.

## Data Availability

Not applicable.
